# Changes in body surface temperature reveal the thermal challenge associated with catastrophic moult in captive gentoo penguins

**DOI:** 10.1242/jeb.247332

**Published:** 2024-06-12

**Authors:** Agnès Lewden, Tristan Halna du Fretay, Antoine Stier

**Affiliations:** ^1^ Université de Brest - UMR 6539 CNRS/UBO/IRD/Ifremer, Laboratoire des sciences de l'environnement marin – IUEM, Rue Dumont D'Urville, 29280 Plouzané, France; ^2^ Université de Strasbourg, CNRS, IPHC UMR 7178, F-67000 Strasbourg, France; ^3^ Department of Biology, University of Turku, FI-20014 Turku, Finland

**Keywords:** Thermal challenge, Moult, Thermoregulation, Penguin, Global warming

## Abstract

Once a year, penguins undergo a catastrophic moult, replacing their entire plumage during a fasting period on land or on sea-ice during which time individuals can lose 45% of their body mass. In penguins, new feather synthesis precedes the loss of old feathers, leading to an accumulation of two feather layers (double coat) before the old plumage is shed. We hypothesized that the combination of the high metabolism required for new feather synthesis and the potentially high thermal insulation linked to the double coat could lead to a thermal challenge requiring additional peripheral circulation to thermal windows to dissipate the extra heat. To test this hypothesis, we measured the surface temperature of different body regions of captive gentoo penguins (*Pygoscelis papua*) throughout the moult under constant environmental conditions. The surface temperature of the main body trunk decreased during the initial stages of the moult, suggesting greater thermal insulation. In contrast, the periorbital region, a potential proxy of core temperature in birds, increased during these same early moulting stages. The surface temperature of the bill, flipper and foot (thermal windows) tended to initially increase during the moult, highlighting the likely need for extra heat dissipation in moulting penguins. These results raise questions regarding the thermoregulatory capacities of penguins in the wild during the challenging period of moulting on land in the current context of global warming.

## Teaser

**Highlighted Article:** Gentoo penguins are probably over-insulated during part of the moult when the two feather layers co-occur, which may challenge their thermoregulatory capacity in the context of climate change.

## INTRODUCTION

In birds, feathers have many functions, including flight, thermal insulation, communication (with plumage coloration; e.g. Bortolotti, 2006) and tactile sensation (Cunningham et al., 2011). Plumage provides thermal insulation for endothermic birds, helping them to maintain a high core body temperature (Prinzinger et al., 1991). Indeed, the feather layers trap air above the skin (Dawson et al., 1999) and plumage colour and the microstructure of plumage elements (Wolf and Walsberg, 2000) reduce conductive, convective and radiative heat loss between the bird and the outside environment (e.g. Calder and King, 1974; Bakken, 1976; Wolf and Walsberg, 2000). This is especially true in aquatic birds such as penguins that show a high density of downy and contour-feathers, increasing water resistance (Pap et al., 2017; Osváth et al., 2018). Penguins have a thick and morphologically specialized plumage (Rutschke, 1965; Williams et al., 2015) providing 80–90% of the insulation requirements (Le Maho et al., 1976; Le Maho, 1977) that enable them to exist in the harshest climates of Antarctica. It is therefore important that penguins are able to maintain high quality plumage (Jenni and Winkler, 2020) through moult: the replacement of old and damaged feathers by new ones (Humphrey and Parkes, 1959).

**Fig. 1.**
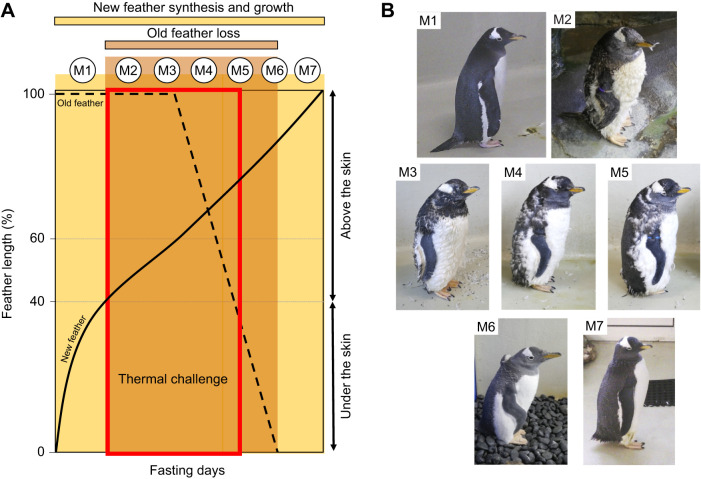
**Identification of the seven main moulting stages (M1–M7) characterized during the gentoo penguin moult in captivity.** (A) A schematic representation of the penguin moult, showing the new feathers (straight line) grow beneath the outer skin layer until 40% of their total length. Between 40% and 60% of new feather growth, penguins have a double feather layer, old (dashed line) and new. These two feather layers could lead to a thermal challenge for heat dissipation (red box). After 60% of new feather growth, the old feathers start to fall off. Adapted from Groscolas and Cherel (1992). (B) During moult, visual plumage changes can be noticed and characterized into seven different stages: M1, uniform old plumage; M2, ‘pop-corn’ (superposition of old and new immature feathers); M3, first fall of old plumage; M4, 25% of old feathers fallen; M5, 50% of old feathers fallen; M6, 75% of old feathers fallen; and M7, uniform new plumage, corresponding to the end of the monitoring. See Materials and Methods for more details.

The moult of penguins is described as ‘catastrophic’ (Davis and Darby, 2012) and occurs once a year during a fasting period on land or on sea-ice, where the heat conductance of air is 25 times lower than that of water (de Vries and van Eerden, 1995) and where seabirds may be particularly vulnerable to heat stress (Chambers et al., 2011). During this time, individuals replace their entire plumage in two overlapping stages, with the synthesis of new feathers preceding the loss of old feathers (Groscolas and Cherel, 1992; Fig. 1A). New feathers begin to grow under the skin until they reach 40% of their size, when they emerge through the skin. At 40–60% of new feather growth, old feathers remain attached to the new feathers and at this stage birds simultaneously have two feather layers (Fig. 1A). The old feathers then fall off, reducing thermal insulation until the new feathers finish growing (Groscolas and Cherel, 1992; Fig. 1A). Moult is an energetically costly period for penguins (Croxall, 1982; Adams and Brown, 1990), despite their low level of activity while fasting on land (Cherel et al., 1994). Indeed, metabolic rate increases by a factor of 1.3 and 1.5 in king penguins (*Aptenodytes patagonicus*; Cherel et al., 1994) and in little penguins (*Eudyptula minor*; Baudinette et al., 1986), respectively. During this fasting period, macaroni penguins (*Eudyptes chrysolophus*) and rockhopper penguins (*Eudyptes chrysocome*) lose 44% and 45% of their body mass, respectively, during a 25 day moult period (Brown, 1986). Similarly, king penguins and emperor penguins (*Aptenodytes forsteri*) lose approximately 45% of their body mass in 30 days, with a peak of daily body mass loss during the final stage of feather loss (Groscolas, 1978; Cherel et al., 1988).

While Groscolas and Cherel (1992) suggested thermal insulation decreased during the loss of old feathers, the preceding overlay of the new and the old feathers could increase the overall thermal insulation of plumage. Metabolic heat production increases during moult as a result of feather synthesis and increased peripheral blood flow to grow the feather (Baudinette et al., 1986; Cherel et al., 1994). During the earlier stages of the moult, when penguins may have a greater thermal insulation due to the double layer of feathers, penguins may face a thermal challenge (Fig. 1A) by being less able to efficiently dissipate metabolic heat, potentially leading to a rise in core body temperature. To investigate this hypothesis, we measured surface temperatures of captive gentoo penguins (*Pygoscelis papua*) using thermal imaging during the entire moulting period. The captive conditions allowed us to measure individuals throughout the full moult period at a uniform air temperature without the effects of solar radiation, wind and/or precipitation. We thus studied how body surface temperatures varied during moult-related changes in physiology and physical state of insulation (i.e. feather growth) independently of environmental conditions. We measured the surface temperature of old and new plumage to represent well-insulated body regions, the periorbital region as a potential proxy of core temperature (e.g. Gauchet et al., 2022), and the surface temperature of the bill, flipper and foot, which correspond to thermal windows (i.e. poorly insulated body areas under vascular control of blood circulation; Tattersall et al., 2009; McCafferty et al., 2013; Lewden et al., 2020). Specifically, we predicted that when penguins possess two simultaneous feather layers (moulting stages M2–M5; see below and Fig. 1) there would be a decrease of plumage surface temperature, an increase in surface temperature of the thermal windows and a potential rise in the temperature of the periorbital region.

## MATERIALS AND METHODS

### Study site

Thirty-one gentoo penguins, *Pygoscelis papua* (Forster 1781) were studied in captivity at Océanopolis^©^ aquarium, Brest, France. Individuals were identified by a coloured plastic ring on the right flipper, and divided into two groups, the first of 18 individuals (7 males and 11 females) and the second of 13 individuals (6 males and 7 females). Individuals were maintained indoors within the only available thermoneutral zone estimates for the species – that is, between 8 and 15°C (Taylor, 1985; Wilson et al., 1998) – in two separate enclosures with similar conditions, i.e. permanent access to free water, unfed during the moulting period and with the same number of enclosure cleaning and animal keeper visits. The lighting programme adopted by the aquarium includes a monthly variation in artificial light, with exposure varying between 13 and 10 h of light per day during our experiment. In addition, some windows allow natural ambient light to penetrate the enclosure without direct exposure to solar radiation. To confirm the absence of direct solar radiation, we measured surface temperature of black and white, old and new plumage, as it is well known that solar heating differs according to coat colour (Cena and Clark, 1973; Benesch and Hilsberg, 2003; McCafferty, 2007). During measurement sessions, air temperature (*T*_a_) and relative humidity (RH) were measured using a weather station Kestrel^®^ 5400 Heat Stress Tracker. The synchronization between the weather station and the thermal camera was carried out prior to the study and checked before each measurement session. The wet-bulb temperature (*T*_w_) was then calculated according to eqn 1 in Stull (2011), to take into account the cooling effect of higher humidity. The enclosures showed a relatively stable *T*_w_ during the study period (from 30 July to 20 October 2022), with a temperature range between 7.20 and 12.56°C in the first group (group 1) and between 9.19 and 14.20°C in the second group (group 2). However, we measured a small but significant difference between groups/enclosures, with a higher *T*_w_ in group 2 (mean±s.e.m. of 10.82±0.39°C) compared with group 1 (9.38±0.28°C) (*P*<0.005). Similarly, the ground surface temperature (*T*_ground_) in contact with the penguins’ feet was higher in group 2 (14.65±0.13°C) compared with group 1 (12.93±0.09°C) (*P*<0.0001). Moult lasted 14.0±0.66 days per individual in group 1 and 12.8±0.97 days per individual in group 2, without a significant difference between groups (*P*=0.85).

### Moult

Penguin surface temperatures were measured once a day in the morning, with a mean of 11.75 (range 2–25) measurements per individual. To track the progress of the moult, seven moult stages were characterized (Fig. 1B) ranging from uniform old plumage (M1) to uniform new plumage (M7) and assigned by the same observer (A.L.) during data collection. The intermediate stages were ‘pop-corn’ during which individuals carried two feather layers giving them a puffy appearance (Fig. 1B; M2). The ‘first fall’ stage corresponds to the advanced pop-corn stage with the first fall of old feathers visible (Fig. 1B; M3). The ‘25%’ stage corresponds to the acceleration of old feather loss, with at least 25% of the trunk having lost its old plumage and thus presenting a new, ‘immature’ plumage (Fig. 1B; M4). The ‘50%’ stage corresponds to the peak of the moult, with 50% of the trunk showing two layers of plumage and 50% exposing the new, so not yet fully grown out, plumage (Fig. 1B; M5). At the ‘75%’ stage (Fig. 1B; M6), individuals had lost most of their old plumage and the new plumage, while not yet fully grown, visibly increased in volume.

### Thermal image collection and analysis

One or two thermal pictures of left or/and right profiles were taken per day of measurement from the same angle (Tabh et al., 2021) at a distance of ca. 1 m, with each body area being larger than 10 times the spot size (Playà-Montmany and Tatersall, 2021) of 0.65 mm, with a FLIR E96 thermal camera (640×480 pixels). For each bird, profile pictures were defined as right (ringed side) or left (non-ringed) side. We hypothesized that the ringed flipper might show a higher surface temperature induced by inflammation linked to ring friction. Emissivity was set to 0.98 (Whittow, 1986; Monteith and Unsworth, 2013) whereas *T*_a_ and RH were set for each picture using Flir ThermaCAM Researcher Professional 2.10 software. Bill, flipper and foot areas were delineated by tracing a polygon around the edge to extract the mean surface temperature of each area (hereafter *T*_bill_, *T*_flipper_, *T*_foot_, respectively). Mean *T*_ground_ was extracted using a standard square size (877 pixels^2^) situated just below the feet. Head was also delineated, and the maximum surface temperature of this area was extracted corresponding to the periorbital region (hereafter *T*_eye_; Jerem et al., 2018). As the loss of old plumage on the trunk was not symmetrical on both sides, we could not determine a representative trunk surface temperature from the profile pictures. Therefore, we calculated a surface temperature representative of the entire trunk (hereafter *T*_trunk_) using the old and the new plumage surface temperature (*T*_old_ _plumage_ and *T*_new_ _plumage_, respectively) according to the percentage of feather loss as follows:
(1)

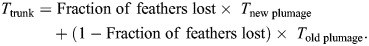
*T*_old_ _plumage_ and *T*_new_ _plumage_ corresponded to the mean surface temperature of a standard square size (438 pixels^2^) positioned on a uniform patch of each type of plumage. When individuals were in old plumage (M1), in pop-corn (M2) and in first fall (M3) stages, *T*_trunk_ corresponded to *T*_old_ _plumage_. When individuals were in new plumage stage (M7), *T*_trunk_ corresponded to *T*_new_ _plumage_. For the other moulting stages (M4, M5 and M6), *T*_trunk_ was calculated using Eqn 1 determined from the cover of the entire trunk circumference to weight the proportion of each plumage type.

### Statistical analysis

The relationship between four temperature areas (i.e. bill, periorbital, flipper and trunk) and moult stages was examined using linear mixed models (LMM) with a Gaussian distribution, including surface temperature as the response variable, with moult stage (M1 to M7), group (1 or 2) and sex (male or female) as explanatory variables, and penguin ID as a random intercept, to control for repeated measures and *T*_w_. For *T*_foot_, *T*_ground_ replaced *T*_w_ in the model considering the large surface area of the foot in contact with the ground (i.e. conductive heat loss). Indeed, the linear relationship between *T*_w_ and *T*_foot_ (*R*^2^=0.02, *P*=0.004) was markedly weaker than the relationship between *T*_ground_ and *T*_foot_ (*R*^2^=0.38, *P*<0.0001). Normality of model residuals was checked visually and did not reveal deviation from Gaussian distribution. The effect of plumage colour (black or white) on old and new plumage surface temperatures was initially investigated using an ANOVA, but dropped from final models because, as expected in the absence of solar radiation, we did not find any significant difference in surface temperature between black and white plumage in either old or new plumage (*P*>0.80 in both cases). Similarly, we did not find any significant effect of the coloured plastic ring on *T*_flipper_ and therefore excluded this variable from our final statistical models (*P*=0.99).

Differences between specific moult stages were investigated using Tukey's honestly significant difference (HSD) *post hoc* tests. Statistical analyses were performed using JMP^®^ v. 13 (SAS Institute Inc., Cary, NC, USA) and results are reported as means±s.e.m. unless otherwise specified.

## RESULTS

Body surface temperatures were positively related to *T*_w_ or *T*_ground_ (Table 1). The variable ‘group’ was only significant for *T*_trunk_ (*P*<0.0001) with a lower *T*_trunk_ measured in the group exposed to the slightly colder environment (i.e. group 1). Females had a slightly higher *T*_eye_ than males (31.02±0.11 and 30.71±0.10°C, respectively; *P*=0.023). Group and sex did not explain any significant variation in *T*_bill_, *T*_flipper_ and *T*_foot_ (Table 1). However, variation in all body surface temperatures was significantly influenced by moult stage (Table 1).

**Table 1.**
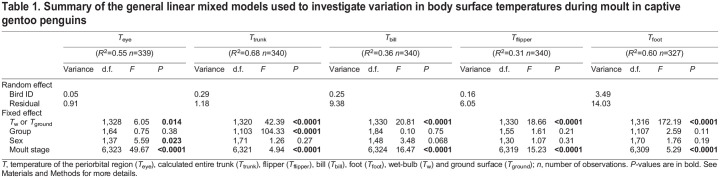
Summary of the general linear mixed models used to investigate variation in body surface temperatures during moult in captive gentoo penguins

**Fig. 2.**
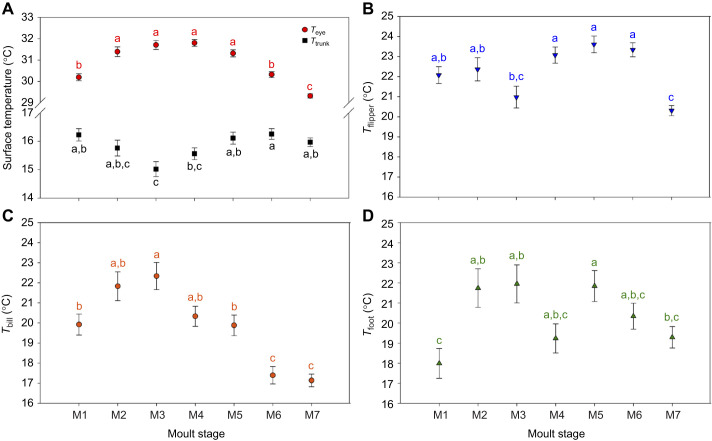
**Mean surface temperatures during the seven moulting stages.** Least-squared mean values for the temperature of the periorbital region (*T*_eye_) and the trunk (*T*_trunk_) (A), the flipper (*T*_flipper_; B), the bill (*T*_bill_; C) and the foot (*T*_foot_; D) are shown. Values that do not share the same letter are significantly different from each other (*post hoc* Tukey's HSD test; *P*<0.05), means are presented ±s.e.m. (*N*=27 individuals, *n*=327–340 observations), and results of linear mixed models are reported in Table 1.

**Fig. 3.**
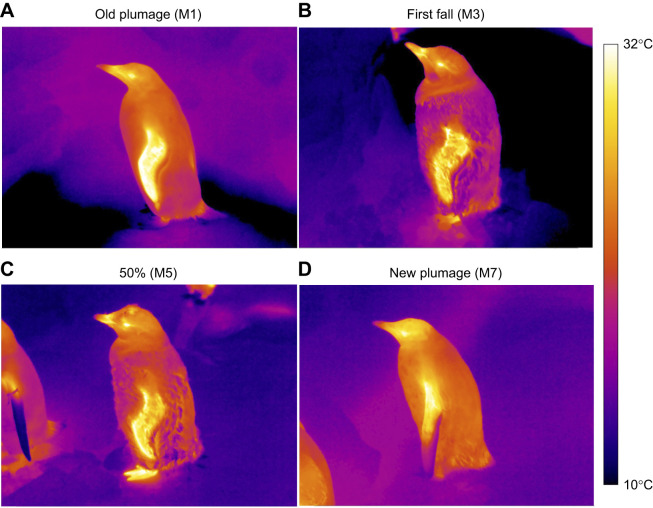
**Visual comparison of four gentoo penguins measured during moult by thermal imaging.** Individuals were measured throughout the moult including M1 (A), M3 (B), M5 (C) and M7 (D). The images illustrate a lower *T*_trunk_ at first fall (M3), lower *T*_flipper_ in new plumage (M7) compared with other stages, higher *T*_bill_ at first fall (M3) than in new plumage (M7), and higher *T*_foot_ during moult (M3 and M5) than before and at the last stage of the moult (M1 and M7).

*T*_eye_ significantly increased from the old plumage (M1) to the pop-corn stage (M2), and stayed elevated until the 50% moult stage (M5; Fig. 2A). *T*_eye_ then decreased at 75% of the moult (M6; to a level similar to that at the old plumage stage, M1), and was lowest at the new plumage stage (M7; Fig. 2A). *T*_trunk_ showed an initial drop from the old plumage stage (M1) to the first fall stage (M3; Fig. 3), and then increased back to its initial level as the moult progressed further towards the new plumage stage (M7; Fig. 2A). *T*_trunk_ at the new plumage stage (M7; 15.93±0.14°C) did not significantly differ from that at the old plumage stage (M1; 16.48±0.21°C; *P*=0.15) (Figs 2A and 3).

*T*_flipper_ had the highest temperature at stage 25%, 50% and 75% of the moult (M4–6; Fig. 2B). *T*_flipper_ at those stages was higher than at the first fall and new plumage stages (M3 and M7; all *P*<0.04; Fig. 3) but did not significantly differ from that at the old plumage and pop-corn stages (M1 and M2; all *P*>0.30). *T*_flipper_ was 1.77°C colder at the new plumage stage compared with old plumage stage (M7 and M1; *P*=0.0002; Fig. 3).

*T*_bill_ significantly increased from the old plumage to first fall stage (M1 to M3) and decreased thereafter (Fig. 2C), with a maximum difference of −5.20°C between the first fall and the new plumage stages (M3 and M7; *P*<0.0001; Fig. 3). *T*_bill_ was also lower at the new plumage stage (M7; 17.13±0.31°C) than at the old plumage stage (M1; 19.92±0.52°C; *P*<0.0001).

*T*_foot_ initially increased from the old plumage until the first fall stage (M1 to M3; Fig. 3), and remained slightly elevated until the end of the monitoring period (i.e. new plumage, M7; Fig. 2D). However, there was no significant difference in *T*_foot_ between the new plumage (M7; 19.29±0.53°C) and old plumage stages (M1; 17.99±0.74°C; *P*=0.62).

## DISCUSSION

Our study investigated the effect of moulting on the surface temperature of captive gentoo penguins. This stage is characterized by a fasting period during which metabolic rate increases (Baudinette et al., 1986; Cherel et al., 1994) while body insulation is heavily modified through full replacement of plumage (Groscolas and Cherel, 1992). Our results showed that at early stages of the moult, when individuals have two feather layers (stages M2, M3 and M4; Figs 1 and 2), plumage insulation was elevated as shown by the lowest *T*_trunk_ (Figs 2 and 3), while the surface temperatures of the thermal windows (bill, flipper and foot) and the periorbital region were generally elevated at these stages (Fig. 2). This effect was maintained until approximately 50% of the moult was completed (M5), after which surface temperatures of non-insulated body regions started to decrease (Figs 2 and 3), such that the mean surface temperatures at the new plumage stage (M7) were significantly lower (except for *T*_foot_) than at the start of the moult (Fig. 2).

Early moulting stages in penguins may therefore provide a thermal challenge for penguins to dissipate extra heat. Moult is energetically costly (Baudinette et al., 1986; Cherel et al., 1994) through maintaining peripheral blood flow for dermal perfusion to sustain feather synthesis. Simultaneously with this higher heat production, we found that the potential for heat dissipation is likely to be reduced by the additional insulation resulting from the combination of newly growing and old feathers, as shown by the tendency of *T*_trunk_ to decrease at pop-corn (M2), first fall (M3) and 25% of moult (M4) stages (Fig. 2). Correspondingly, uninsulated or less insulated body areas from the bill, flipper and foot exhibited the opposite pattern (Figs 2 and 3), with an increase of surface temperature allowing greater heat loss through radiation and convection to the surroundings. Under our captive experimental conditions, birds were measured within their thermoneutral zone (between 8 and 15°C; Taylor, 1985), which theoretically eliminates any changes in metabolic rate associated with thermoregulation. Thus, our study highlights that blood flow to thermal windows may help to compensate for greater insulation and heat production associated with feather synthesis, to maintain a stable core body temperature during early moulting stages. Moreover, with similar patterns measured in feathered flippers and in unfeathered bill and feet, our results support the thermal dissipation function of peripheral blood flow and not only the role of increased peripheral blood flow for feather synthesis during the moult. As *T*_eye_ is relatively well correlated with core temperature in some bird species such as chickens (Cândido et al., 2020), budgerigars (*Melopsittacus undulates*; Ikkatai and Watanabe, 2015) and wild red-footed boobies (*Sula sula*; Gauchet et al., 2022), we hypothesize that *T*_eye_ may also be a proxy of core temperature in penguins, even if this relationship has not yet been validated. With this assumption, our results would suggest that core body temperature increased during the early stage of moult (+1.61°C of *T*_eye_ between old plumage and 25% stage, M1 to M4; Fig. 2A). This idea is supported by an increase of ca. 0.8°C in core temperature during moult in yellow-eyed penguin (*Megadyptes antipodes*; Farner, 1958). However, blood flow to the eyes, impacting *T*_eye_, has also been shown to play a role in reducing brain temperature in pigeons (*Columba livia*; Pinshow et al., 1982), and has been suggested as a heat sink in ostriches (*Struthio camelus*; Fuller et al., 2003) or to maintain/enhance visual acuity in great tits (*Parus major*) when stressed (Winder et al., 2020). Consequently, without core body temperature measurements or previous validation of the relationship between core body temperature and *T*_eye_ in penguin species, it is not possible here to formally assess whether the increased heat dissipation through thermal windows we observed was sufficient to maintain a stable core body temperature, or whether the observed increase in *T*_eye_ could reflect the inability of penguins to thermoregulate without a rise in core temperature.

Interestingly, the thermal challenge to dissipate heat described here at thermoneutrality seems likely to be specific to penguins, due to the accumulation of this double insulation. Indeed, most studies in birds measured the opposite pattern, with an increase of 30–60% in thermal conductance during moult (Lustick, 1970; Dietz et al., 1992), inducing for instance a rise of the lower critical temperature in long-eared owl (*Asio otus*; Wijnandts, 1984). In pinnipeds, moult represents the renewal of hair but in a few species of seals the outer skin layer is also shed (Ling, 1968, 1972). In harbour seals (*Phoca vitulina*) with relatively little accumulation of old fur, individuals show increased thermal costs through greater heat loss (Paterson et al., 2012). In Antarctica Weddell seals (*Leptonychotes weddellii*), Walcott et al. (2020) measured that the energetic cost of thermoregulation doubled during moult, with an increase of 25% in heat loss in early moult stages. Importantly, no thermal windows were detected in that study, suggesting that individuals were unlikely to overheat (Walcott et al., 2020). Similar results were obtained in southern elephant seals (*Mirounga leonina*), with an increase of 1.8 times the resting metabolic rate during their catastrophic moult of hair and epidermis, with body surface temperature decreasing throughout the moult (Paterson et al., 2022), the latter suggesting an energy-saving strategy. Moreover, elephant seals also showed aggregation behaviour only during their moult on land, with movement and habitat selection dependent on windchill and solar radiation (Chaise et al., 2018), suggesting a strategy to reduce heat loss and minimize energy costs (Chaise et al., 2019). In contrast, *Pygoscelis* penguins do not exhibit specific aggregation behaviour or habitat selection during moulting, to the best of our knowledge, and remain mainly inactive in both wild and captive conditions (Penney, 1967; Cherel et al., 1994). In contrast, penguins found in temperate regions show behavioural adaptations to heat by using shaded sites such as burrows (Frost et al., 1976; Luna-Jorquera, 1996; Simeone et al., 2004; for review, see Colombelli-Négrel and Iasiello, 2023). In gentoo penguins, whereas foraging habitats at sea have been studied over the past few decades (e.g. Williams and Rothery, 1990; Lescroël and Bost, 2005; Miller et al., 2010; Camprasse et al., 2017), habitat selection on land remains understudied, except during the breeding season (Quintana et al., 2000; Quintana, 2001). It is worth noting that ambient temperature during moulting periods in the wild may remain relatively low, but solar radiation may be high and lead to high operative temperatures. The thermal challenge from changes in physiology and plumage insulation during moult suggests that penguins may be required to select habitats to avoid high heat gain from the environment (e.g. solar radiation) in the current context of global warming.

Body surface temperatures during the new plumage stage (M7) did not reach the initial temperature measured in old plumage (M1) and these temperatures were either lower (*T*_bill_, *T*_eye_ and *T*_flipper_) or similar (*T*_foot_, *T*_trunk_) at the end of the moult. Firstly, moult may have started before a visible change in plumage (i.e. pop-corn stage, M2). In this case, the old plumage stage (M1) could already correspond to an early stage of moult (i.e. growth of new feathers below the skin; Fig. 1), with a higher metabolism, rather than a pre-moult stage as initially considered in this study. Secondly, the new plumage stage (M7) corresponds here to termination of the old feather loss and potentially not to the end of new feather growth. The ‘immature’ new feathers could be less insulated than the full-length feathers, allowing heat loss without a specific need to maintain blood flow to the thermal windows. Conversely, the insulative quality of old feathers at the end of their life (M1), i.e. at the start of the moult, is likely to be compromised and thus could induce increased heat loss. Finally, as moulting is also associated with the progression into a more advanced fasting stage, it is possible that at the new plumage stage (M7), penguins could use peripheral vasoconstriction as an energy-saving strategy (Kooyman et al., 1976; Ponganis et al., 2001, 2003; Tattersall et al., 2016). This may explain the decreases we observed in *T*_bill_, *T*_eye_ and *T*_flipper_.

In summary, our study shows that under nearly constant environmental conditions, moulting gentoo penguins increase the surface temperature of poorly insulated regions (thermal windows) (Figs 2 and 3) to dissipate extra heat. In the wild, the thermal challenge of carrying two feather layers could be greater as a result of solar radiation and an increase of air temperature in the current context of global warming (Ainley et al., 2010; Gorodetskaya et al., 2023), but this remains to be investigated in wild penguin populations.
